# Intelligence, Correspondence, &c.

**Published:** 1825-10-01

**Authors:** 


### INTELLIGENCE, CORRESPONDENCE, &c.

*Anatomie Vivante; or, Skeleton importation Company.*

These are the days of speculation! One of the most profitable, has been that of the Skeleton Importation Company. It appears that some of our half-pay captains, whose former duty it was, to "eat Frenchmen alive," have lately changed their occupation, (in conformity with these piping times of peace,) to that of picking the bones of a French skeleton; and good picking they have! Between three and four hundred of the hydra-headed but little-witted multitude of John Bulls—or rather John Gulls, have run daily to Pall Mall to get their half-crown share or sight of the living skeleton! In short, no Frenchman ever before excited such curiosity on these shores—except Napoleon himself. The living skeleton may therefore, in this respect, be considered as a second *Bony*-parte. The motto of the Company, over their office in Pall Mall, is very appropriate: *viz.* "*Praise God Barebones.*"

And after all, this wretched emblem of mortality does not present a single thing, from head to foot, that *ought* to excite, or *can* possibly gratify the laudable curiosity of saint, of savage, or of sage. The pictorial representations that have been paraded through the streets, pasted to the walls, and pourtrayed in the print-shops, are the reverse of *naked* truths—they are so many *naked* lies; and the whole is a scene of gross exaggeration on the part of the Company, and gullibility on that of the public.

We saw this meagre importation before he was exhibited to the public, in company with one of the first medical characters of this metropolis, and, it was our joint opinion that he would excite no interest whatever among the faculty of this country. The great expectation was that medical men would give any sum of money to see the wonderful structure and functions of a living skeleton; but the fact is, that there is nothing more to be seen than a man irregularly emaciated—the emaciation not being half so great as what we every day see in hospitals and other sanctuaries of sickness. He has considerable malformation of the chest, which was probably an original cause of the defective nutrition; but we strongly suspect that the emaciation is not entirely natural, and that this miserable being augments his natural meagreness by voluntary abstinence. Be this as it may, there is nothing in the case that can lead to the elucidation of any point in physiology or pathology.

---

*Dr. Heiniker.*

In the first volume of this series, page 486, *et. seq.* our readers will perceive a short notice of a paper on the climate of Madeira, by Dr. Heiniker, in which the reviewer regrets that Dr. H. should have treated the subject of phthisis with more levity than was suitable to such a subject. It appears, from a note in our respected cotemporay, the Medical Repository for September, (we have not yet seen Dr. H's. remonstrance) that the author "has felt aggrieved at Dr. Johnson's remarks." Now it is really very hard, and certainly unjust, that the conducting Editor of a Medical Review should be singled out by name, and assigned as the writer of every article in an extensive periodical journal. If Dr. Heiniker or the reader will please to refer to the volume and page, he will see by the very next article in succession, that Dr. Johnson was himself prostrate on the bed of sickness at the time; and Dr. J. can conscientiously aver that he never wrote a line of the critique, nor was he capable even of correcting the press during that quarter. This will convince Dr. H. that he is not indebted to Dr. Johnson for the animadversions on his paper, and it will probably induce other authors to be a little more charitable towards the editor, and not to place the sins of all the writers in this Journal to his individual account. The proper and just mode is surely to remonstrate with the *reviewer* and not with the Editor. On the present occasion, Dr. Johnson is truly grieved that any expression in the Journal should have hurt the feelings of any gentleman—and most of all the feelings of "an unresisting invalid." It is not the general character of this Journal to trample on the meek and truckle to the proud. Whenever it permits the *feelings of the heart* to influence the *judgment of the head*, it is far more inclined

"parcere subjectis et debellare superbos."

On recurring, however, to the critique in question (and we request our readers to re-peruse it) we really think that the animadversions could only have irritated a mind that was morbidly sensible to moral impressions.—Dr. H. is merely accused of having too great a flow of animal spirits, and of being able, like some of our most antient and sapient philosophers, to laugh at his own corporeal infirmities.—When we next visit Funchal, we shall be happy to ascend with Dr. H. to the mountain Convent, and crack a bottle of Madeira, together with many jokes, while viewing the picturesque and romantic scenes, which that delightful spot commands. Till then—vive valeque.

---

Dr. Hartman of Leipzig has in the Press a Translation into German of Dr. Chisholm's Manual of Tropical Climates, and also of Dr. Johnson's 3d. Edition of "the Influence of Tropical Climates on European Constitutions."

---

*Sea Water Baths.*

We are very friendly to the extension of baths of every description in this country—and especially in this metropolis. We therefore heard, with much pleasure, that, among the numerous companies which the speculative age we live in has brought forth, there was one forming for the purpose of erecting baths in different parts of London, and supplying them with water from the ocean, at an expence considerably under that at which the same luxuries and medicinal agencies can now be procured. We consider it the duty and interest of the medical profession to encourage this undertaking, as it will, when carried into effect, afford them much facility in the application of a measure of the very highest importance in the practice of their avocations. In our next Number we hope to be able to lay more of the particulars before our readers.

---

Dr. GORDON SMITH will commence a course of lectures on FORENSIC MEDICINE and MEDICAL POLICE in the beginning of October. For a prospectus, containing particulars, apply to the Medical Booksellers.

---

N. B.—The Review of Dr. John Mason Good's *Second Edition* of the Study of Medicine will, we hope, appear in our next. We may state, in the mean time, that this Edition has received great improvements.

